# Contribution of SecDF to *Staphylococcus aureus *resistance and expression of virulence factors

**DOI:** 10.1186/1471-2180-11-72

**Published:** 2011-04-12

**Authors:** Chantal Quiblier, Annelies S Zinkernagel, Reto A Schuepbach, Brigitte Berger-Bächi, Maria M Senn

**Affiliations:** 1 Institute of Medical Microbiology, University of Zurich, Gloriastr. 32, 8006 Zurich, Switzerland; 2 Division of Infectious Diseases and Hospital Epidemiology, University Hospital Zurich, University of Zurich, Raemistr. 100, 8091 Zurich, Switzerland; 3 Surgical Intensive Care, University Hospital Zurich, University of Zurich, Raemistr. 100, 8091 Zurich, Switzerland

## Abstract

**Background:**

SecDF is an accessory factor of the conserved Sec protein translocation machinery and belongs to the resistance-nodulation-cell division (RND) family of multidrug exporters. SecDF has been shown in *Escherichia coli *and *Bacillus subtilis *to be involved in the export of proteins. RND proteins can mediate resistance against various substances and might be of relevance in antimicrobial therapy. The role of RND proteins in *Staphylococcus aureus *has not yet been determined.

**Results:**

Markerless deletion mutants were constructed to analyze the impact of the so far uncharacterized RND proteins in *S. aureus*. While the lack of Sa2056 and Sa2339 caused no phenotype regarding growth and resistance, the *secDF *mutant resulted in a pleiotropic phenotype. The *secDF *mutant was cold sensitive, but grew normally in rich medium at 37°C. Resistance to beta-lactams, glycopeptides and the RND substrates acriflavine, ethidium bromide and sodium dodecyl sulfate was reduced. The *secDF *mutant showed an aberrant cell separation and increased spontaneous and Triton X-100 induced autolysis, although the amounts of penicillin-binding proteins in the membrane were unchanged. The impact of *secDF *deletion on transcription and expression of specific virulence determinants varied: While coagulase transcription and activity were reduced, the opposite was observed for the autolysin Atl. A reduction of the transcription of the cell wall anchored protein A (*spa*) was also found. The accumulation of SpA in the membrane and lowered amounts in the cell wall pointed to an impaired translocation.

**Conclusions:**

The combination of different effects of *secDF *deletion on transcription, regulation and translocation lead to impaired cell division, reduced resistance and altered expression of virulence determinants suggesting SecDF to be of major relevance in *S. aureus*. Thus SecDF could be a potential target for the control and eradication of *S. aureus *in the future.

## Background

*Staphylococcus aureus *is a frequent colonizer of the human body as well as a serious human pathogen. It is known for its adaptability to diverse environments. It can cope with stress factors and acquire resistances to antibiotics thus rendering treatment difficult. *S. aureus *can cause a wide range of infections, mainly due to an impressive arsenal of virulence determinants comprising cell surface components and excreted factors interacting with the host system. Transport of proteins to the cell surface and secretion to the extracellular space is mediated through different transport systems [1] of which the general protein secretion system Sec plays a prominent role in protein export and membrane insertion.

Sec-mediated translocation has best been studied in *Escherichia coli *and is catalyzed by the essential SecYEG protein complex (reviewed in [2]). The motor ATPase SecA or a translating ribosome is believed to promote protein export by driving the substrate in an unfolded conformation through the SecYEG channel. The accessory SecDF-YajC complex facilitates protein export and membrane protein insertion efficiency in vivo [3], possibly via the control of SecA cycling [4]. The large exoplasmic loops of the integral membrane proteins SecD and SecF have been shown to be required for increasing protein translocation by a yet unknown mode of action [5]. While *secDF *disruption leads to a cold-sensitive phenotype and defects in protein translocation [6], the absence of YajC, which interacts with SecDF, causes only a weak phenotype [7]. SecYEG has been shown to interact with the SecDF-YajC complex [8]. YidC, a protein that is proposed to mediate membrane integration and the assembly of multimeric complexes, can also interact with SecDF-YajC to take over SecYEG-dependent membrane proteins [9].

Data on the *S. aureus *Sec system is scarce: SecA and SecY have been shown to be important, respectively essential, for growth by using antisense RNA [10]. Deletion of *secG *resulted in an altered composition of the extracellular proteome, which was aggravated in a *secG secY2 *double mutant [11]. Deletion of *secY2 *alone, which together with *secA2 *belongs to the accessory Sec system [12], did not show any effect on protein translocation. As in the Gram-positive bacterium *Bacillus subtilis*, in *S. aureus *the accessory SecD and SecF proteins are fused to form a single protein (Sa1463), which was identified in membrane vesicles [13]. However, the chromosomal organization in *S. aureus *resembles the one of *E. coli*, with *yajC *lying immediately upstream of *secDF*. Furthermore, SecDF was identified in a surface-exposed peptide epitope screen by using a cell shaving technique [14] and expression was found to be slightly higher in a COL *sigB *deletion mutant [15]. SecDF is postulated to be essential in *S. aureus *according to a mutagenic screen [16].

SecDF belongs to the resistance-nodulation-cell division (RND) family of multidrug export pumps, that is conserved and widely distributed in all three major kingdoms of life [17]. RND proteins have a wide substrate specificity and diverse functions ranging from the efflux of noxious host derived substances, such as bile salts by *E. coli *[18] to the involvement of eukaryotic efflux pumps in cholesterol homeostasis in humans [19]. Multiple antibiotic resistance can be associated with these exporters, as they often recognize a broad range of substrates, thereby diminishing drug accumulation in the cell [20,21]. *S. aureus *possesses two additional uncharacterized RND proteins, namely Sa2056, located downstream of the essential *femX *[22], and Sa2339 (MmpL homologue).

## Results

### Construction of the *rnd *mutants

To evaluate the role and impact of the RND proteins in *S. aureus*, markerless deletion mutants were constructed in the sequenced and well-characterized clinical strain Newman. SecDF, Sa2056 and Sa2339 were found to be dispensable, as we obtained null mutants by allelic replacement of the corresponding genes using the pKOR1 system of Bae et al. [23]. The mutants were confirmed to have generally retained genome stability and to carry the desired modification in the corresponding locus as described in methods.

Deletion of *sa2056 *and *sa2339 *had no apparent effect on *S. aureus *when evaluating growth and resistance properties (data not shown), suggesting that they may be important under other conditions than applied in this study. The following report is therefore focused on the *secDF *mutant and its phenotype.

### Transcription of *secDF *and growth phenotype of the *secDF *mutant

**Figure 1:**
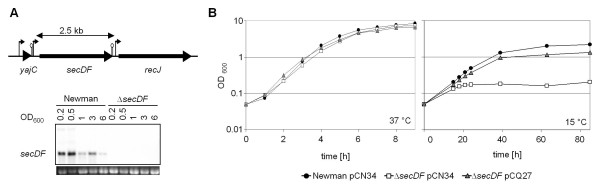
**Growth characteristics of the *secDF *mutant**. **(A**) Genetic context of *secDF *in *S. aureus *and Northern blot analysis of *secDF *transcription during growth. Predicted promoter and terminators are depicted. Ethidium bromide-stained 16S rRNA is shown as an indication of RNA loading. **(B) **Growth of Newman and the *secDF *mutant carrying the empty vector pCN34, and of the complemented mutant *secDF *pCQ27 in LB broth at 37°C and 15°C, respectively.

Transcription of *secDF *was monitored from early exponential to early stationary phase and found to result mainly in a monocistronic mRNA. *secDF *was strongest transcribed during early growth phase and declined towards stationary phase (Figure 1A). As expected, no transcripts were detected in the *secDF *deletion mutant. Transcriptional profiles were restored in the mutant by introducing the complementing plasmid pCQ27, containing the *secDF *gene from Newman with its endogenous promoter (data not shown).

**Figure 2:**
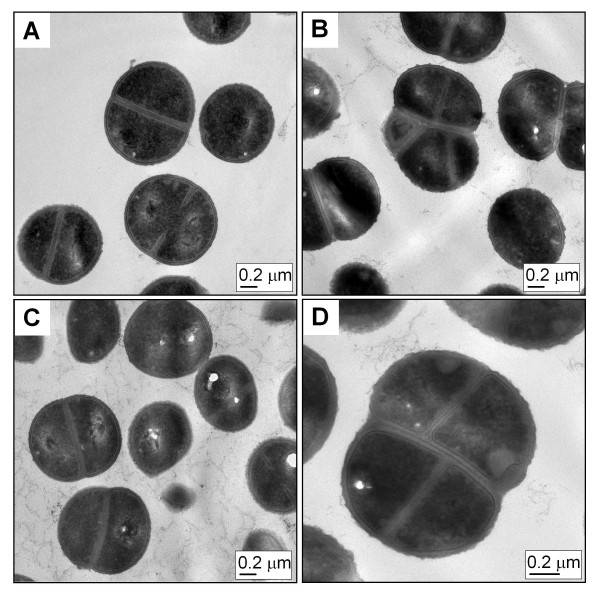
**Cell morphology**. TEM pictures from thin sections of strains **(A) **Newman pCN34, **(B and D) **Δ*secDF *pCN34 and **(C) **Δ*secDF *pCQ27 during exponential phase (OD_600 _0.5).

At 37°C no significant difference was observed when comparing the growth curves of the wild type strain Newman and the mutant (Figure 1B). However, colonies of *secDF *mutants were smaller on blood agar compared to the wild type (83% ± 5.1 of the wild type's cross section). TEM pictures were prepared from exponentially growing cells. In contrast to the wild type (Figure 2A) and the complemented mutant (Figure 2C), displaying normally shaped cells with a maximum of one septum, the *secDF *mutant had difficulties in separating daughter cells (Figure 2B and 2D). This resulted in clusters with sometimes multiple and wrongly placed septa. At least 400 cells per strain were analyzed, showing that 20.4 ± 8.7% of the mutant cells could not divide correctly whereas this was only the case in 0.3 ± 0.7% for the wild type and 0.9 ± 1.3% for the complemented mutant.

As *secDF *knock out mutants in *B. subtilis *and *E. coli *show a cold sensitive phenotype [6,24], growth of the *S. aureus secDF *mutant was tested at 15°C. The temperature drop affected the *secDF *mutant approximately after two generations, causing a notably reduced growth rate with a subsequent halt in growth after 24 h. The plasmid pCQ27, but not the empty vector pCN34, significantly restored growth at 15°C (Figure 1B).

### Increased susceptibility of the *secDF *mutant towards RND-substrates, β-lactam and glycopeptide antibiotics

**Figure 3:**
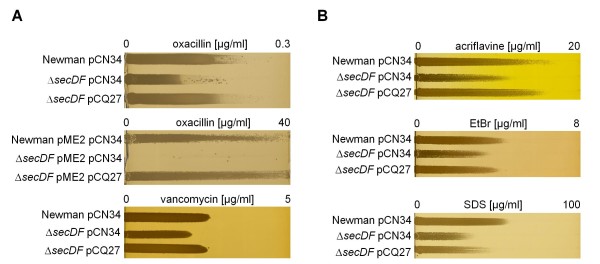
**Effect of *secDF *inactivation on resistance profiles**. **(A) **Gradient plates with increasing concentrations of β-lactam and glycopeptide antibiotics. Oxacillin was tested with methicillin sensitive and methicillin resistant strain Newman, the latter carrying the plasmid pME2 containing the *mecA *gene. **(B) **Gradient plates with increasing concentrations of the RND substrates acriflavine, ethidium bromide and SDS.

Since multidrug resistance can be mediated unspecifically by RND exporters [21,25], we characterized the resistance profile of the *secDF *mutant by testing several different classes of antibiotics and typical RND-substrates [26,27]. The *secDF *mutant showed increased susceptibility to the RND substrates acriflavine, ethidium bromide and sodium dodecyl sulfate (SDS) on gradient plates (Figure 3). Furthermore, a distinct increased susceptibility to the β-lactam oxacillin and the glycopeptide vancomycin was observed (Figure 3). The reduction of oxacillin resistance was even more apparent in the presence of *mecA*, the gene encoding the penicillin binding protein 2a (PBP2a), mediating methicillin resistance, as shown for the methicillin resistant *S. aureus *(MRSA) strain pair Newman pME2 and Newman *secDF *pME2 (Figure 3) [28]. Reduction of oxacillin resistance in MRSA by *secDF *inactivation was confirmed in strains of different genetic backgrounds or SCC*mec *types, such as the clinical isolate CHE482 [29] and RA2 [30] or RA120 [31] (data not shown). The complementing plasmid pCQ27 was able to restore the wild type resistance levels.

**Figure 4:**
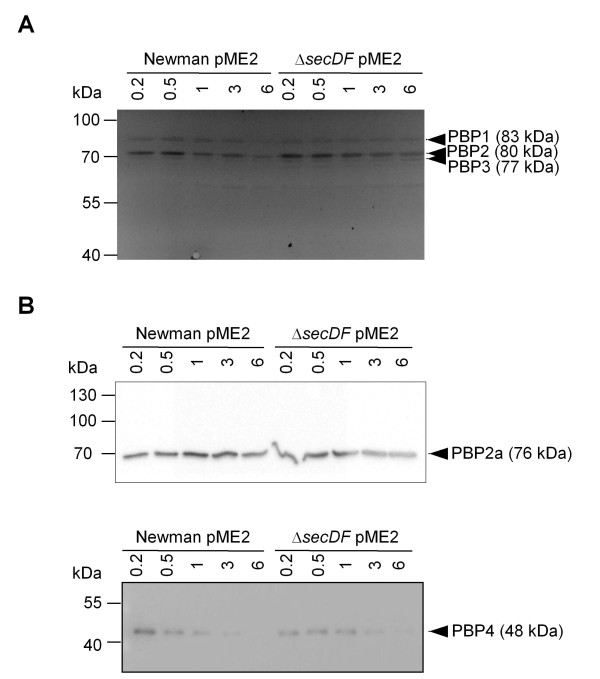
**PBP expression over growth**. Strain Newman pME2, carrying *mecA*, and its *secDF *mutant were cultivated in LB and samples collected at the indicated OD_600 _were used to prepare membrane fractions. **(A) **Membranes were incubated with the fluorescent penicillin analogue Bocillin-FL. Bands corresponding to PBPs 1-3 are indicated. **(B) **Western blot analysis of membrane fractions using antibodies against PBP2a and PBP4, respectively.

Of the four endogenous *S. aureus *PBPs, PBP1 and PBP2 are essential, and reducing their expression lowers methicillin resistance even in the presence of the low β-lactam affinity PBP2a in MRSA [32,33]. As the Sec-system can promote protein insertion into the cytoplasmic membrane, we determined whether the reduced oxacillin resistance of the *secDF *mutant may be related to altered PBP amounts and/or subcellular localization. Staining cell membranes with the fluorescent penicillin-derivative Bocillin-FL [34] showed no major difference of PBP1-3 content in wild type MRSA background or corresponding *secDF *mutants (Figure 4A). However, Bocillin-FL staining did not allow the detection of the Sec-type signal peptide containing PBP4 [1] of approximately 48 kDa, or to distinguish the exogenous PBP2a in the Newman background (Figure 4A and 4B), possibly due to low protein levels or overlap, respectively. Western blots revealed comparable PBP2a and PBP4 amounts in the membrane fraction throughout growth, irrespective of the presence of SecDF (Figure 4B).

### Increased autolysis and hydrolysis in the *secDF *mutant

**Figure 5:**
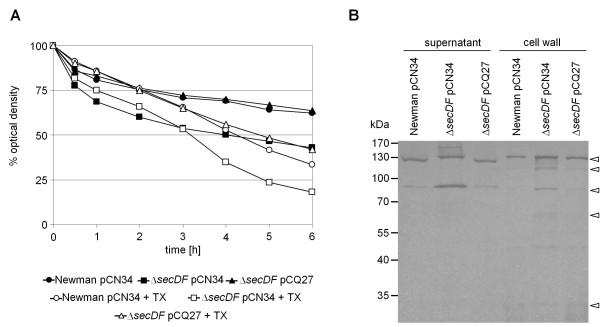
**Autolysis and zymogram**. **(A) **Spontaneous and Triton X-100 (TX) induced autolysis was measured over time. **(B) **Autolysin zymography of protein extracts from supernatant and cell wall was performed using SDS-10% PAGE supplemented with *S. aureus *cell wall extract as a substrate. Dark bands show hydrolyzed cell wall and are indicated by triangles. Based on the work of Schlag et al. bands were assigned as follows in decreasing order: Pro-Atl (~130 kDa); Atl (~115 kDa); Atl-amidase (~84 kDa) or part of the propeptide (62-65 kDa); Sle1/Aaa (~33 kDa) [35].

Apart from functional PBPs, correct separation of daughter cells requires the controlled action of autolysins and hydrolases, many of which are Sec-dependent [1]. We therefore tested spontaneous and Triton X-100 induced autolysis to determine if the inability of *secDF *mutants to separate correctly was due to altered expression of autolytic activities. Both, spontaneous and Triton X-100 induced autolysis of the *secDF *mutant were increased in comparison to the wild type or the complemented mutant (Figure 5A).

To determine whether wild type and mutant bacteria produced different levels of hydrolases, their activity was analyzed in concentrated supernatant and cell wall extracts (Figure 5B). In the supernatant of the mutant, high molecular mass bands matching different forms of the major *S. aureus *autolysin Atl [35], were expressed similarly (>130 kDa, pro-Atl) or even stronger (~84 kDa, PP-AM) compared to the wild type and the complemented mutant (Figure 5B). Interestingly, the >130 kDa band migrated at a slightly higher position in the mutant, corresponding to the height of the pro-Atl band in the cell wall fractions, where the mutant showed overall stronger hydrolytic bands than wild type or complemented mutant.

### Deletion of *secDF *leads to altered expression of virulence factors

We qualitatively assessed the amount of various Sec-dependent *S. aureus *virulence factors, such as coagulase, hemolysin and protease activities, as well as of the immunomodulatory protein SpA to determine whether they were affected in the *secDF *mutant as well.

**Figure 6:**
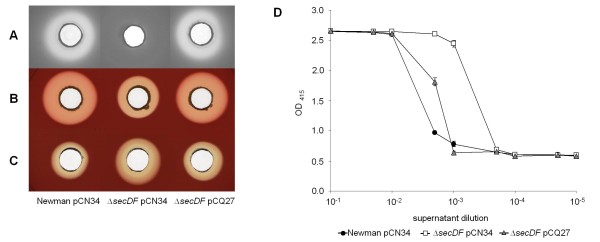
**Proteolysis and hemolysis of sessile and planktonic bacteria**. Proteolytic and hemolytic activity was determined qualitatively by agar diffusion assay on skim milk, respectively sheep blood agar. Hemolytic activity was measured in diluted sheep blood. **(A) **Skim milk agar and **(B) **sheep blood agar with sessile bacteria. **(C) **Sheep blood agar with sterile-filtered supernatants of stationary phase planktonic bacteria. **(D) **Release of hemoglobin by stationary phase supernatants of planktonic bacteria. Representative data of three independent experiments are shown with standard deviations of technical triplicates.

Supernatant from Newman and the complemented *secDF *mutant coagulated rabbit plasma after 30 min, whereas the *secDF *mutant required 90 min, suggesting production of slightly reduced coagulase levels. Extracellular proteolytic activity seemed to be absent in the *secDF *mutant, even after five days of incubation, whereas cultures from Newman and the complemented mutant showed distinct proteolytic halos on skim milk agar after 72 h (Figure 6A). Hemolysis activity was tested by a similar agar diffusion assay as used for protease activity determination. Overnight cultures, or sterile-filtrated culture supernatants, were dispensed into holes on sheep blood agar. Newman and the complemented *secDF *mutant showed the same extent of hemolysis. In the *secDF *background hemolysis was reduced when bacteria grew on the rim of agar holes (Figure 6B), but was increased when the hemolytic activity of sterile supernatant from liquid cultures was tested (Figure 6C and 6D). Sessile or planktonic growth affects regulatory mechanisms, which can alter the expression of virulence factors such as Hla [36,37]. Here we found that the deletion of *secDF *had divergent effects on hemolysin expression depending on the growth conditions, most likely by affecting regulatory processes.

SpA is one of the proteins predicted to be attached to the cell wall by sortase following export [38]. SpA levels were determined in subcellular fractions during growth by Western blot analyses.

**Figure 7:**
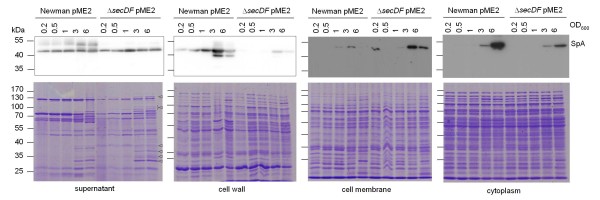
**Subcellular localization of SpA**. Expression and localization of SpA was monitored in the Newman pME2 background during growth. Upper panels show Western blots of SpA. Longer exposure times were required for detection of SpA in cell membrane and cytoplasm. Bottom panels show Coomassie stained gels. Bands of stronger expression in the mutant are indicated by triangles.

Compared to the wild type, SpA levels were reduced in the cell wall and the cytoplasmic fraction, but slightly increased in the cell membrane fraction of the *secDF *mutant (Figure 7). The SpA levels were similar in the supernatant. Processed SpA has a molecular weight of approximately 51 kDa in strain Newman as estimated by Western blot analysis of wild type and Δ*spa *protein extracts (Additional file 1: Figure S1). Larger bands (~53 kDa) in the wild type supernatant fraction most likely represent SpA still attached to cell wall fragments. Thus, SpA translocation and/or processing seemed to be affected by the *secDF *deletion, a phenotype that could be complemented by introducing pCQ27 (data not shown).

Surprisingly, secreted SpA amounts were fairly constant despite this translocation defect. Also in the wild type, SpA levels in the supernatant were constant, whereas the amount of cell wall-bound SpA increased during growth, suggesting constant shedding of this protein.

### Transcriptional analyses of virulence factors reveal regulatory changes in the *secDF *mutant

**Figure 8:**
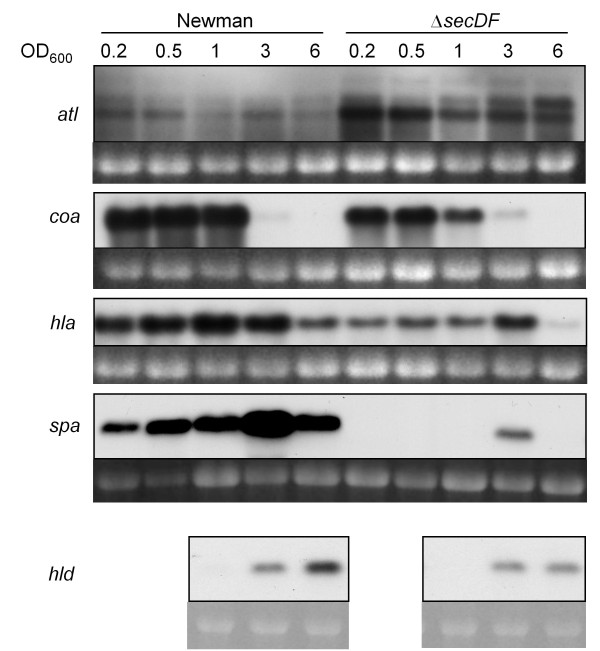
**Transcription of virulence factors**. *atl*, *coa*, *hla*, *spa *and *hld *transcription was monitored over growth in strains Newman and Δ*secDF*. Ethidium bromide-stained 16S rRNA is shown as an indication of RNA loading.

To determine whether the altered protein levels in the *secDF *mutant reflected also the mRNA level, transcription of *atl *(~3.8 kb), *coa *(~1.9 kb), *hla *(~1 kb) *hld *(~0.5 kb) and *spa *(~1.6 kb) were examined at different growth phases. *atl *transcription was elevated in the mutant during the entire growth (Figure 8) which is in agreement with the increased hydrolytic activities observed (Figure 5B). Transcription of *coa *sharply decreased after OD_600 _of 1. Slightly lower transcription levels were seen for *coa *in the *secDF *mutant (Figure 8), which is in line with our findings for its coagulation properties. As Newman carries a prophage in the *hlb *gene [39] and the gamma toxin is inhibited by sulfonated polymers in agar [40], we only looked at the transcription of the genes encoding α and δ toxins. *hla *amounts in the mutant were reduced compared to the wild type (Figure 8). The transcription pattern of *hld*, contained in the major regulatory RNAIII, had a tendency to being slightly reduced in the mutant but still showed a growth phase dependent expression, starting at OD_600 _3 (Figure 8, data was assessed for the relevant ODs 1, 3 and 6). A striking difference was observed for the *spa *transcription, which in the wild type increased over growth with a peak at OD_600 _3, but was drastically reduced in the *secDF *mutant (Figure 8). These findings were in contrast to the observed higher hemolytic activities and SpA amounts in the supernatant (Figure 6 respectively 7), which either suggests increased stability or reduced degradation of these proteins in the *secDF *mutant.

## Discussion

Efflux pumps play an important role in *S. aureus *resistance, virulence and pathogenicity. Yet the impact of the RND family of efflux pumps in staphylococcal resistance and fitness is still open (reviewed in [41]). To our knowledge, this is the first study to evaluate their role in *S. aureus*.

We found SecDF to contribute probably in part indirectly to resistance against several substances, including β-lactams and glycopeptides, making it an interesting target for increasing the efficacy of these standard antibiotics. In contrast Sa2056 and Sa2339 seemed not to be required for growth and resistance under the conditions tested. Banerjee et al. recently had found a conservative amino acid mutation in Sa2056 in a high-level β-lactam resistant *mecA*-negative strain [42]. However in that strain PBP4 and Sa0013 were also mutated and the exact reason for the observed resistance phenotype was not identified.

Resistance against cell wall active antibiotics and cell separation is dependent on a tightly balanced regulation of cell wall synthetic and hydrolytic enzymes, including their timely localization to the septum [43,44]. The amount of PBPs 1-4 and PBP2a was apparently not influenced, suggesting that other factors important for cell division and β-lactam resistance were affected. The increased hydrolytic activity in the *secDF *mutant may explain the observed differences in cell wall production and separation. Overproduction of a hydrolase has been observed to affect formation of the FtsZ-ring in *Mycobacterium tuberculosis *[45]. This cytoskeleton structure recruits the other cell division proteins to the site of future cell separation. A similar indirect effect in the *secDF *mutant might have lead to an incorrect localization of the cell division machinery, including PBPs (for a general review see [46]), thereby causing reduced resistance against the cell wall active antibiotics oxacillin and vancomycin. The difference in Atl processing might have impeded proper cell separation in addition.

Like *E. coli *and *B. subtilis secDF *mutants [6,24], the *S. aureus secDF *mutant displayed a cold-sensitive phenotype. In *E. coli *and *B. subtilis *SecDF has furthermore been shown to participate in membrane integration and secretion of proteins [6,24,47]. In *S. aureus *many physiological functions were affected by the *secDF *deletion. Analysis of the secretion of classical *S. aureus *virulence factors containing a Sec-type signal peptide revealed a complex picture. Coagulase and proteases were reduced in the supernatant in the *secDF *mutant. However, hemolysin activity under planktonic growth conditions was increased in the mutant, as was the case for (unprocessed) hydrolases, indicating that the *secDF *deletion did not lead to an overall reduction, but to an altered secretion and processing of proteins. In contrast hemolysin activity was reduced during sessile growth indicating that the deletion of *secDF *may have effects on overall metabolism.

SpA seemed to be impaired in reaching its destined subcellular localization. In the *secDF *mutant SpA accumulated in the membrane, was reduced in the cell wall fraction but was found in increased amounts in the supernatant. Altered secretion and processing of SpA might be due to impaired cell wall anchoring by the membrane protein sortase. However, Mazmanian et al. have shown that the extracellular enterotoxin B fused to the sorting signal of SpA accumulates in the cytoplasm and to a lesser extent in the membrane in a sortase mutant [48]. Thus, SpA might migrate by an alternate mechanism into the supernatant, circumventing linking to the peptidoglycan.

A similar divergent effect on protein secretion as we observed in the *secDF *mutant was found in a *secG *mutant. There SpA was found in increased amounts in the exoproteome, despite unaffected transcription [11]. In contrast, we found deletion of *secDF *to change mRNA levels for many of the analyzed genes, such as *atl*, *coa*, *hla*, *hld *and *spa*. The lack of *secDF *therefore seems to have a different impact on virulence factor expression than *secG*, influencing, most likely indirectly, transcription in addition to translocation. The absence of SecDF could especially cause a defective or reduced membrane insertion of sensor proteins belonging to one of the numerous *S. aureus *two component systems contributing to virulence factor regulation and to adaptations to different growth conditions (reviewed in [49,50]). The reduced *hld *levels in the mutant suggests that the *secDF *deletion affected at least one two component system by impairing signaling via the *agr *quorum sensor [51].

This study and the work of Sibbald et al. [11] once more demonstrate that protein and mRNA levels do not necessarily correlate. Specific regulation at the protein level has been shown for certain transcription factors in *S. aureus *[52,53]. Such a control of protein stability via chaperones and proteases might exist as well for virulence factors. Interestingly, in *E. coli*, *secY*, *yidC *and *secD *mutants were shown to induce the Cpx system, which up-regulates the expression of factors involved in folding and proteolysis in response to abnormal proteins in the outer membrane, the periplasmic space or the plasma membrane [54]. The induction of similar systems in the *S. aureus secDF *mutant due to clogging of the membrane, as suggested by the increased amounts of SpA in this compartment, could be an additional factor influencing protein stability and lead to the partially incoherent mRNA and protein levels, as seen for *hla*, *hld *and *spa *during planktonic growth.

## Conclusions

This work provides evidence that although *secDF *is dispensable in *S. aureus*, its deletion leads to a pleiotropic phenotype. Lack of SecDF affected cell separation, resistance and virulence factor expression showing that this conserved RND protein plays a major role in the important human pathogen *S. aureus*. Thus SecDF could be a potential therapeutic target rendering *S. aureus *more susceptible to the currently available antibiotics.

## Methods

### Bacterial strains and growth conditions

Strains and plasmids used in this study are listed in Table 1. Bacteria were grown aerobically at 37°C in Luria-Bertani broth (LB) (Difco) where not mentioned otherwise. Good aeration for liquid cultures was assured by vigorously shaking flasks with an air-to-liquid ratio of 4 to 1. Ampicillin 100 [μg/ml], anhydrotetracycline 0.2 [μg/ml], chloramphenicol 10 [μg/ml], kanamycin 50 [μg/ml] or tetracycline 10 [μg/ml] were added to the media when appropriate. Phage 80αalpha was used for transduction. Where nothing else is mentioned, experiments were repeated at least twice and representative data are shown.

**Table 1:** Strains and plasmids used in this study

Strain	Relevant genotype or phenotype	Ref. or source
S. aureus		
Newman	Clinical isolate (ATCC 25904), *rsbU*^+^	[64]
RN4220	NCTC8325-4 r^- ^m^+^	[65]
CQ33	NewmanΔ*sa2056*	This study
CQ39	Newman pME2, Tc^r^, Mc^r^	This study
CQ65	NewmanΔ*sa2339*	This study
CQ66	NewmanΔ*secDF*	This study
CQ69	NewmanΔ*secDF *pME2, Tc^r^, Mc^r^	This study
CQ85	Newman pCN34, Km^r^	This study
CQ86	Newman pCN34 pME2, Km^r^, Tc^r^, Mc^r^	This study
CQ87	NewmanΔ*secDF *pCN34, Km^r^	This study
CQ88	NewmanΔ*secDF *pCN34 pME2, Km^r^, Tc^r^, Mc^r^	This study
CQ89	NewmanΔ*secDF *pCQ27, Km^r^	This study
CQ90	NewmanΔ*secDF *pCQ27 pME2, Km^r^, Tc^r^, Mc^r^	This study
E. coli		
DH5α	Cloning strain, [F-Φ80*lac*ZΔM15 Δ(*lac*ZYA-*arg*F)U169 *rec*A1 *end*A1 *hsd*R17 (rk-, mk+) *pho*A *sup*E44 *thi*-1 *gyr*A96 *rel*A1 λ-]	Invitrogen

**Plasmid**	**Relevant genotype or phenotype**	**Reference or source**
pCN34	*S. aureus-E. coli *shuttle vector, pT181-*cop-wt repC aphA-3 *ColE1 Km^r^	[56]

pCQ27	pCN34 derivative carrying *secDF *and its promoter (Newman), Km^r^	This study

pCQ30	pKOR1 derivative carrying 1 kb fragments of the region up- and downstream of *sa2056 *amplified from Newman, ligated together with EcoRI and recombined at the *attP *sites, Ap^r^, Cm^r^	This study

pCQ31	pKOR1 derivative carrying 1 kb fragments of the region up- and downstream of *sa2339 *amplified from Newman, ligated together with HindIII and recombined at the *attP *sites, Ap^r^, Cm^r^	This study

pCQ32	pKOR1 derivative carrying 1 kb fragments of the region up- and downstream of *secDF *amplified from Newman, ligated together with HindIII and recombined at the *attP *sites, Ap^r^, Cm^r^	This study

pKOR1	*E. coli-S. aureus *shuttle vector used to create markerless deletions; *repF*(Ts) *cat attP ccdB ori *ColE1 *bla *P*_xyl_*/*tetO secY570*, Ap^r^, Cm^r^	[23]

pME2	pBUS1 derivative carrying *mecA *and its promoter (COLn), Tc^r^, Mc^r^	[28]

Abbreviations are as follows: Ap^r^, ampicillin resistant; Cm^r^, chloramphenicol resistant; Km^r^, kanamycin resistant; Mc^r ^methicillin resistant; Tc^r^, tetracycline resistant.

### Construction of mutants and complementation plasmid

In-frame markerless deletions of *sa2056 *(NWMN 2'384'867-2'388'051), *sa2339 *(NWMN 2'696'046-2'698'531) and *secDF *(NWMN 1'706'584-1'708'866) from the chromosome of *S. aureus *Newman (accession number NC_009641) was performed using pKOR1 [23] yielding single mutants CQ33, CQ65 and CQ66, respectively. Correct deletion was confirmed by PCR and by sequencing. Furthermore, strain stability was confirmed by pulsed field gel electrophoresis of total genome SmaI digests [55].

To complement the *secDF *mutant, *secDF *with its endogenous promoter was amplified from *S. aureus *strain Newman with primers listed in additional file 2 table S1.

The amplified region was ligated into the SalI/BamHI restriction sites of pCN34, a low copy (20-25 copies/cell) *E. coli*-*S. aureus *shuttle vector [56]. The junction region was sequenced as a control. The resulting plasmid pCQ27 was electroporated into RN4220 with subsequent transduction into the strains of interest.

To construct MRSA strains, the plasmid pME2, containing the *mecA *promoter and gene from strain COLn [28], was either electroporated or transduced into the strains selected.

Promoter predictions were performed by BPROM http://linux1.softberry.com/berry.phtml. Rho-independent transcriptional terminators were retrieved from the CMR terminator list http://cmr.jcvi.org/tigr-scripts/CMR/CmrHomePage.cgi.

### Transmission electron microscopy (TEM)

Cells were grown to exponential phase, harvested at OD_600 _0.5 and fixed for one hour in 2.5% glutaraldehyde in phosphate buffered saline (PBS) pH 7.4. Electron microscopy was performed by the Center for Microscopy and Image Analysis, University of Zurich.

### Resistance profiles

For qualitative susceptibility comparisons, bacterial suspensions of McFarland 0.5 were swapped across LB agar plates containing antibiotic gradients and incubated at 35°C for 20-24 h. Glycopeptides were tested on Brain Heart Infusion (BHI) (Difco) agar with a bacterial suspension of McFarland 2 [57].

### Spontaneous and Triton X-100 induced autolysis

Cells were grown to an OD_600 _of 0.7, pelleted by centrifugation and washed with 0.85% NaCl. The cells were then resuspended in 0.01 M Na-phosphate buffer pH 7 and the OD_600 _was adjusted to 0.7. After splitting the cultures, 0.01% Triton X-100 (Fluka) or an equal volume of PBS pH 7 was added. Cultures were incubated at 37°C and the decrease of OD_600 _was measured.

### Zymographic analyses

Cultures were grown to an OD_600 _= 0.7, centrifuged and the filtered supernatants (pore size 0.45 μm, TPP) stored at - 20°C until further use. The cell wall peptidoglycan was digested in SMM buffer (0.5 M sucrose, 0.02 M maleate, 0.02 MgCl_2 _pH 6.5) supplemented with 72 μg/ml lysostaphin and 2 mM phenylmethylsulfonyl fluoride (PMSF) [38]. Cell wall containing supernatant was separated from the protoplasts and stored at - 20°C until further use. Protein concentrations were measured by Bradford assay (BioRad).

Twenty μg of protein from each fraction was separated by SDS-10% polyacrylamide gel electrophoresis (PAGE) containing cell wall extract of heat-inactivated (1 hour at 100°C in 4% SDS) *S. aureus *(end concentration OD_600 _= 6). The gel was washed twice for 15 min in dH_2_O and incubated for 18 h at 37°C in 0.1 M Na-phosphate buffer pH 6.8. Afterwards the gel was incubated for 3 min in staining solution (0.4% methylene blue, 0.01% KOH, 22% EtOH) and destained in cold water for several hours. Murein hydrolase activities produced clear bands.

### Coagulase test

Overnight cultures were pelleted at full speed, 0.5 ml supernatant was transferred into fresh tubes and 2 mM PMSF was added. The supernatants were normalized to an OD_600 _of 1 of the original culture with PBS. 0.1 ml supernatant was added to 0.25 ml reconstituted rabbit plasma (BBL Coagulase Plasmas, BD) and incubated at 37°C. Every 30 min tubes were examined for coagulation.

### Qualitative hemolysis assay

Cells were grown overnight in Todd-Hewitt (TH) medium [58], which was originally developed for the production of streptococcal hemolysins [59]. To visualize hemolysis production of sessile bacteria, overnight cultures were normalized to an OD_600 _= 1 in PBS pH 7.4. Fifty μl was dispensed into 5 mm wide holes punched into 5% sheep blood agar. Plates were incubated overnight at 37°C and then stored at 4°C. To determine hemolysis in liquid media, the overnight cultures grown in TH medium were normalized to the same OD_600 _with PBS and pelleted for 10 min at 5'900 g. The supernatant was filtered (pore size 0.22 μm, TPP) and 140 μl added to the holes in sheep blood agar. Plates were incubated as above.

### Quantitative hemolytic activity

Cells were grown for 24 h in TH medium and normalized with PBS pH 7.4 to the same OD_600_. After pelleting the cells, the filtered supernatants (pore size 0.22 μm, TPP) were diluted up to 1:50'000 in TH medium. Sterile sheep blood was treated with 26 mM sodium citrate and 15 mM NaCl and diluted 1:100 in PBS pH 7.4. After washing the erythrocytes four times in PBS pH 7.4, they were resuspended to a dilution of 1:100 in PBS pH 7.4. Five hundred μl of washed erythrocytes were added to 500 μl of the diluted supernatants and incubated for 30 min at 37°C, followed by 30 min at 4°C. Finally the samples were centrifuged for 1 min at 7'000 g and the absorption of hemoglobin in the supernatant was measured at 415 nm [58].

### Determination of protease activity on skim milk agar plates

Skim milk agar plates were prepared as follows: Skim milk (Difco) and Bacto agar (Difco) were dissolved separately in 250 ml dH_2_O, each with an end concentration of 75 g/l and 15 g/l, respectively. After autoclaving for 15 min at 110°C and cooling down to 50°C, the skim milk and Bacto agar solutions were mixed together. Overnight cultures grown in LB broth were normalized to an OD_600 _= 1 with 0.85% NaCl and 50 μl was added into punched holes in skim milk agar. Skim milk agar plates were incubated at 37°C for 24 h and another 96 h at room temperature.

### Transcription analyses

Prewarmed LB broth was inoculated with an overnight culture to an OD_600 _0.05 and incubated at 37°C. Cells were harvested at OD_600 _0.2, 0.5, 1, 3 and 6, centrifuged for 5 min at 20'000 g and 4°C. Cells were immediately snap frozen in liquid nitrogen and stored at - 80°C. Total RNA was extracted as described in [60]. Seven μg RNA was separated in a 1.5% agarose gel containing 20 mM guanidine thiocyanate in 1× TBE [61]. RNA was transferred onto a positively charged nylon membrane (Roche) using the downward capillary transfer method. The blots were hybridized with specific digoxigenin (DIG)-labeled DNA probes (Roche). Primers used are listed in Additional file 2 Table S1.

### Analyses of subcellular protein fractions

Cells were sampled as described for transcription analyses and culture supernatant was collected as described for zymographic analysis. Cells were fractionated basically according to Schneewind et al. [38]. Briefly, cells were digested in SMM buffer supplemented with each 72 μg/ml lysostaphin and lysozyme, 36 μg/ml DNase and 2 mM PMSF. Protoplasts were separated from the cell wall containing supernatant by centrifugation for 4 min at 16'000 g. Protoplasts were resuspended in membrane buffer (0.1 M NaCl, 0.1 M Tris-HCl, 0.01 MgCl_2 _pH 7.5) and lysed by three cycles of freezing in liquid nitrogen/thawing at 20°C. Cell membranes were separated from the cytoplasm by centrifugation for 30 min at 20'000 g and 4°C. Membrane pellets were solubilized in buffer B (25 mM Tris-HCl pH 7.5, 150 mM NaCl, 1 mM MgCl_2_, 30% glycerol) supplemented with 1% Triton X-100 and 0.5% N-lauroylsarcosine, by gently mixing end-over-end at 4°C. Where necessary, protein fractions were concentrated with Amicon Ultra-15, -4 or -0.5 centrifugal filter units (MWCO 10 kDa, Millipore). Cell fractions were kept at - 20°C.

Five μg of protein was separated by SDS-10% PAGEs and either stained with Coomassie Imperial™ Protein Stain (Thermo Scientific) or blotted onto a PVDF-membrane (Immobilon-P, Millipore). For detection of SpA, membranes were blocked with 5% milk powder in PBS and then incubated with goat anti-human IgA conjugated with horseradish peroxidase (HRP, Sigma-Aldrich), 1:10'000 in 0.5% milk powder/PBS, 0.05% Tween 20 (AppliChem). After washing three times with PBS pH 7.4, HRP was detected with SuperSignal West Pico Chemiluminescent substrate (Thermo Scientific). PBP2a was detected as described in [28]. For detection of PBP4, membranes were blocked with 5% milk powder in PBS. Membranes were pre-incubated with 40 μg/ml human IgG in 0.5% milk powder/PBS. Rabbit anti-PBP4 antibodies (1:2000, [62]) and 0.05% Tween 20 were then added. After incubation for 1 h, membranes were washed three times with PBS before addition of goat anti-rabbit IgG-HRP (Jackson ImmunoResearch), 1:10'000 in 0.5% milk powder/PBS/0.05% Tween 20. After washing three times with PBS, HRP was detected as described for SpA. Molecular weights of PBP2a, PBP4 and unprocessed SpA are 76 kDa, 48 kDa and 56.7 kDa, respectively.

### Bocillin-FL staining

Hundert μg of cell membrane fraction were incubated for 30 min at 35°C with Bocillin-FL (Invitrogen) as described by [63] before separation by SDS-7.5% PAGE. Fluorescence was visualized with the FluorChem™ SP imaging system (AlphaInnotech).

## Authors' contributions

CQ carried out construction of strains, phenotypic characterizations, transcription analysis and drafted the manuscript. ASZ and RAS contributed to the growth condition experiments and participated in writing of the manuscript. MMS carried out the Western blot analyses, Bocillin-FL staining and participated in writing the manuscript. BBB coordinated the study and participated in writing of the manuscript. All authors read and approved the final manuscript.

## Supplementary Material

Additional file 1 **Figure S1 - SpA processing in strain Newman**. Western blot analyses of **(A) **subcellular fractions of wild type grown to an OD_600 _of 3 and **(B) **of total extract from overnight cultures of wild type and *spa *mutant using goat anti-human IgA antibodies. Coomassie stained total protein is shown on the right as an indication of loading. SN, supernatant; CW, cell wall; CM, cell membrane; CP, cytoplasm. Click here for file

Additional file 2 **Table S1 - Primers used in this study**. Click here for file
