# Supplementary material for: Contribution of SecDF to Staphylococcus aureus resistance and expression of virulence factors
Source: BMC Microbiol. 2011 Apr 12;11:72. doi: 10.1186/1471-2180-11-72 (PMC3090319; doi:10.1186/1471-2180-11-72)
Supplement: Additional file 1 — Figure S1 - SpA processing in strain Newman. Western blot analyses of (A) subcellular fractions of wild type grown to an OD600 of 3 and (B) of total extract from overnight cultures of wild type and spa mutant using goat anti-human IgA antibodies. Coomassie stained total protein is shown on the right as an indication of loading. SN, supernatant; CW, cell wall; CM, cell membrane; CP, cytoplasm. [file 1471-2180-11-72-S1.PDF]

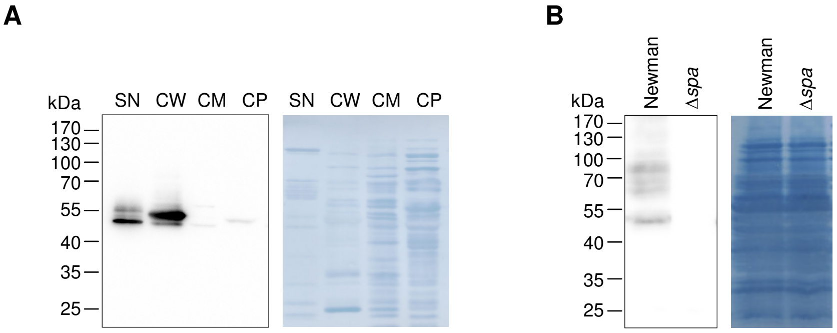

Figure S1 - SpA processing in strain Newman

Western blot analyses of **(A)** subcellular fractions of wild type grown to an OD_{600} of 3 and **(B)** of total extract from overnight cultures of wild type and *spa* mutant using goat anti-human IgA antibodies. Coomassie stained total protein is shown on the right as an indication of loading. SN, supernatant; CW, cell wall; CM, cell membrane; CP, cytoplasm.
