# Supplementary material for: Contribution of SecDF to Staphylococcus aureus resistance and expression of virulence factors
Source: BMC Microbiol. 2011 Apr 12;11:72. doi: 10.1186/1471-2180-11-72 (PMC3090319; doi:10.1186/1471-2180-11-72)
Supplement: Additional file 2 — Table S1 - Primers used in this study. [file 1471-2180-11-72-S2.PDF]

Table S1: Primers used in this study

Primer	Sequence 5'-3'	Use	Reference
atl+	CCAAGGAACCATTGATAAGC	DIG labeling *atl*	This study
atl-	TGATACTGCTAAACCTACGC	DIG labeling *atl*	This study
CQ27	GCATCATTAGGTACAATCGTG	DIG labeling *sa2056*	This study
CQ31	AGTGTGGGGAACATACTTAAGTG	For sequencing junction Δ*sa2056*	This study
CQ61	TGCTTCGGCAATTAGTGTTG	DIG labeling *sa2339*	This study
CQ62	GCCCTTGCTTAGGTATCACG	DIG labeling *sa2339*	This study
CQ63	ATCGCCGCAGCAGTATTATT	DIG labeling *secDF*	This study
CQ65	GCACGCGTTAAATCGTCTTT	DIG labeling *secDF*	This study
CQ77	*GGGGACCACTTTGTACAAGAAAGCTGGGT*CCTGGTTATGCAATCGGCGGTTTG	pCQ32 construction	This study
CQ78	CACAAGCTTACCTCATTATTTACGTATGT	pCQ32 construction	This study
CQ81	*GGGGACCACTTTGTACAAGAAAGCTGGGT*TGCTTCATAATGAACAAGGG	pCQ31 construction	This study
CQ82	TTTAAGCTTAGTATATTGCCTCCTTTTAAAATC	pCQ31 construction	This study

Table S1 continued.

Primer	Sequence 5’-3’	Use	Reference
CQ83	ATAAAGCTTAAAATAACATGTACATGCCTCCGC	pCQ31 construction	This study
CQ84	*GGGGACAAGTTTGTACAAAAAAGCAGGCT*GCTTGATACTTATCATGAGATG	pCQ31 construction	This study
CQ85	ATTAAGCTTTAAAATGAATTAAGCGGTATGTGAAACAATAAAGAG	pCQ32 construction	This study
CQ86	*GGGGACAAGTTTGTACAAAAAAGCAGGCT*GCTAATGATGCGTCATCTAAACGACCTACAGC	pCQ32 construction	This study
CQ90	GAGGTCGGGGATAGATACT	For sequencing junction Δ*sa2339*	This study
CQ91	CTCCCTGCTCTAGTATGTT	For sequencing junction Δ*secDF*	This study
CQ98	ACTGGATCCTTTAAACTAAAATCTTTTCATCGTTCG	*secDF* complementation	This study
CQ100	TCATTGCCGTTCCGCTATGG	For sequencing junction in pCQ27	This study
CQ101	GGGGACCACTTTGTACAAGAAAGCTGGGTCCTGATGTTGAAGTTGATAAAGG	pCQ30 construction	This study
CQ102	TCACGAATTCTCCCCCTCTTTCACTATATTC	pCQ30 construction	This study

Table S1 continued.

Primer	Sequence 5’-3’	Use	Reference
CQ103	TAATGAATTCGCCATAAAAGCGGTCATGATATTG	pCQ30 construction	This study
CQ104	*GGGGACAAGTTTGTACAAAAAAGCAGGCT*CCACAGATATTTTAGAAGTCTACTG	pCQ30 construction	This study
CQ105	GCAAATCAAGTCCTAAATTGAC	For sequencing junction in pCQ27	This study
CQ109	GGTGGTCGACAAGGTACTGTTAAAGCAG	*secDF* complementation	This study
CQ118	GGAGCACGCGAAAGAGTTACG	DIG labeling *coa*	This study
CQ119	CACGGATACCTGTACCAGCATC	DIG labeling *coa*	This study
MS20	AGAAAATGGCATGCACAAAAA	DIG labeling *hla*	[1]
MS21	TGTAGCGAAGTCTGGTGAAAA	DIG labeling *hla*	[1]
MS115	GGCTAGTCCTTTAACCTGTTTC	DIG labeling *sa2056*	This study
spaF	TGTAGGTATTGCATCTGTAA	DIG labeling *spa*	[2]
spaR	AAGTTAGGCATATTCAAGAT	DIG labeling *spa*	[2]

Restriction sites used for cloning are underlined. attB1 and attB2 sites written in italics.

Reference

1. Senn M: **Approach on resistance strategies in *Staphylococcus aureus*: I Cell-membrane associated steps of peptidoglycan synthesis. II Temporal patterns of global regulator in a *hemB* mutant**. *Diss University of Zurich* 2005.

2. McCallum N, Bischoff M, Maki H, Wada A, Berger-Bächi B: **TcaR, a putative MarR-like regulator of *sarS* expression**. *J Bacteriol* 2004, **186**(10):2966-2972.
